# Two-wave modified cardiometabolic index change patterns and incident edentulism in Chinese adults: a longitudinal study from CHARLS

**DOI:** 10.1186/s12903-026-09177-7

**Published:** 2026-08-13

**Authors:** Jin Wang, Fang Li, Junfeng Wu, Ziqi Yang, Yihao Pei, Fance Meng, Kan Xu

**Affiliations:** 1 Medical College of Jinzhou Medical University, Jinzhou, 121000 China; 2 Department of Geriatrics, Zhongshan Hospital (Xiamen), Fudan University, Xiamen, 361015 China; 3 Department of Stomatology, Anting Hospital, No. 204 Changji Road, Jiading District, Shanghai, 201805 China; 4 Department of Geriatrics, Zhongshan Hospital, Fudan University, No. 180 Fenglin Road, Xuhui District, Shanghai, 200032 China

**Keywords:** Dental Care, Preventive Dentistry, Health Behavior, Cardiometabolic health, Longitudinal Studies, Oral Health

## Abstract

**Objectives:**

Complete tooth loss is a late-stage oral health endpoint that may reflect cumulative oral, systemic, behavioral, and social risk processes. We examined whether two-wave patterns of the modified cardiometabolic index (MCMI) from 2011 to 2015 were associated with incident edentulism from 2015 to 2018 among Chinese adults.

**Methods:**

We analyzed participants aged 45 years or older from the China Health and Retirement Longitudinal Study who reported having natural teeth in 2015 and had MCMI measured in 2011 and 2015. Using wave-specific medians, we grouped participants into four 2011–2015 MCMI patterns: stable high, high-to-low, low-to-high, and stable low. Incident edentulism was defined as self-reported complete tooth loss in 2018 among those at risk in 2015. Risk ratios were estimated using modified Poisson regression with robust variance, with stepwise adjustment for sociodemographic and behavioral factors; fully adjusted models additionally included baseline MCMI. Cumulative MCMI and simple change in MCMI were also examined as tertiles.

**Results:**

Among 3,626 adults, 231 developed edentulism by 2018, yielding a 6.4% cumulative incidence. Compared with the stable high pattern, participants who moved from high to low MCMI had a lower risk of incident edentulism in the fully adjusted model (adjusted RR, 0.57; 95% CI, 0.34 to 0.97). The low-to-high and stable low patterns showed no clear associations after adjustment. In parallel analyses, neither cumulative MCMI nor simple change tertiles demonstrated a consistent gradient in edentulism risk.

**Conclusions:**

In this longitudinal CHARLS analysis, a high-to-low MCMI change pattern was associated with lower subsequent risk of incident edentulism compared with persistently high MCMI, whereas cumulative MCMI and absolute change showed no consistent gradient. Because edentulism is a late-stage cumulative endpoint and baseline oral health measures were unavailable, these findings should be interpreted as observational associations rather than evidence of a direct cardiometabolic effect on tooth loss.

**Supplementary Information:**

The online version contains supplementary material available at https://doi.org/10.1186/s12903-026-09177-7.

## Introduction

Edentulism, defined as the complete loss of natural teeth, remains a major oral health challenge in ageing societies. Beyond impaired mastication, it can compromise dietary quality, communication, social participation, and oral health-related quality of life, and it contributes to the global disability burden attributed to oral conditions [1, 2]. In China, population ageing and unequal access to preventive and restorative dental care amplify this challenge. National oral health epidemiological surveys provide a standardized framework for monitoring tooth loss and rehabilitation needs, and findings from the fourth national survey indicate substantial tooth loss in adults, incomplete restoration, and marked geographic disparities in prosthetic rehabilitation [3]. Longitudinal evidence in Chinese middle-aged and older adults further indicates that self-reported edentulism has increased over time and clusters with chronic diseases, suggesting that complete tooth loss often occurs within broader multimorbidity profiles [4]. Edentulism has also been linked to frailty, cognitive impairment, sarcopenia, and mortality in older adults, supporting its relevance as a late-stage oral health endpoint that reflects accumulated biological, behavioral, and social risk processes [5–7].

Cardiometabolic dysregulation provides a plausible systemic context for these associations. Central adiposity, dyslipidemia, hyperglycemia, and elevated blood pressure frequently co-occur and define metabolic syndrome, a state linked to chronic low-grade inflammation and microvascular dysfunction that may impair periodontal homeostasis and tissue repair [8, 9]. Epidemiologic syntheses suggest that metabolic syndrome components are associated with periodontitis across populations, reinforcing shared inflammatory and behavioral risk structures between cardiometabolic disease and oral inflammatory damage [10]. Obesity-related pathways may also operate through bidirectional links between tooth loss and nutritional patterns, with systematic evidence supporting associations between excess adiposity and tooth loss or edentulism [11]. At a mechanistic level, insulin resistance has been associated with periodontal inflammation, and vascular factors such as blood pressure may partly mediate this relationship, highlighting intertwined cardiometabolic and periodontal pathways rather than isolated risk factors [12].

Composite cardiometabolic indices have been developed to summarize these interrelated metabolic components. The cardiometabolic index, derived from lipid ratios and adiposity measures, was introduced to improve discrimination of metabolic risk beyond single markers [13]. Building on this concept, the modified cardiometabolic index (MCMI) incorporates fasting glucose and central adiposity metrics to reflect combined glycemic, lipid, and adiposity burden [14]. Related indices have been associated with metabolic endpoints such as non-alcoholic fatty liver disease, liver fibrosis, and future cardiovascular events, supporting their usefulness as integrated cardiometabolic summaries in epidemiologic studies [15, 16]. For oral health research, repeated assessment of such summaries may help describe whether cardiometabolic burden remains high, declines, increases, or remains low during a defined exposure window.

Despite growing evidence linking cardiometabolic health to oral outcomes, several gaps remain. Higher cardiometabolic index levels have been associated with periodontitis prevalence in US adults [17], systematic reviews indicate that metabolic syndrome is linked to greater tooth loss [18], and studies from Japan and South Korea suggest associations between metabolic syndrome and tooth loss burden [19, 20]. However, many studies rely on single-timepoint exposure assessments or focus on partial tooth loss rather than incident edentulism. Evidence remains limited on whether longitudinal changes in integrated cardiometabolic burden are associated with subsequent complete tooth loss, particularly in Chinese ageing cohorts where both cardiometabolic risk and edentulism are common [4, 21].

In this study, we used longitudinal data from the China Health and Retirement Longitudinal Study (CHARLS) to examine whether two-wave MCMI change patterns between 2011 and 2015 were associated with incident edentulism from 2015 to 2018 among middle-aged and older Chinese adults [22]. We also evaluated cumulative MCMI and absolute MCMI change as alternative exposure summaries. Because edentulism is a late-stage cumulative endpoint, this study was designed to estimate prospective associations across complementary cardiometabolic exposure summaries rather than to establish a direct causal sequence between cardiometabolic change and complete tooth loss.

## Methods

### Study design and data source

This study used data from the China Health and Retirement Longitudinal Study (CHARLS), a nationwide cohort of Chinese adults in midlife and older age [22]. CHARLS was selected because it tracks cardiometabolic biomarkers and oral health over time in a large, population based sample, which allows exposure assessment to precede outcome ascertainment [23, 24]. The CHARLS protocol was approved by the Peking University Ethics Review Committee, and all participants provided written informed consent.

### Study population and analytic cohort

The analysis was organized around two consecutive periods. MCMI was assessed in 2011 and 2015 (Wave 1 and Wave 3), and incident edentulism was evaluated from 2015 to 2018 (Wave 4), which keeps the exposure window ahead of the outcome window. This design ensured that cardiometabolic exposure was characterized before incident edentulism was ascertained.

**Fig. 1:**
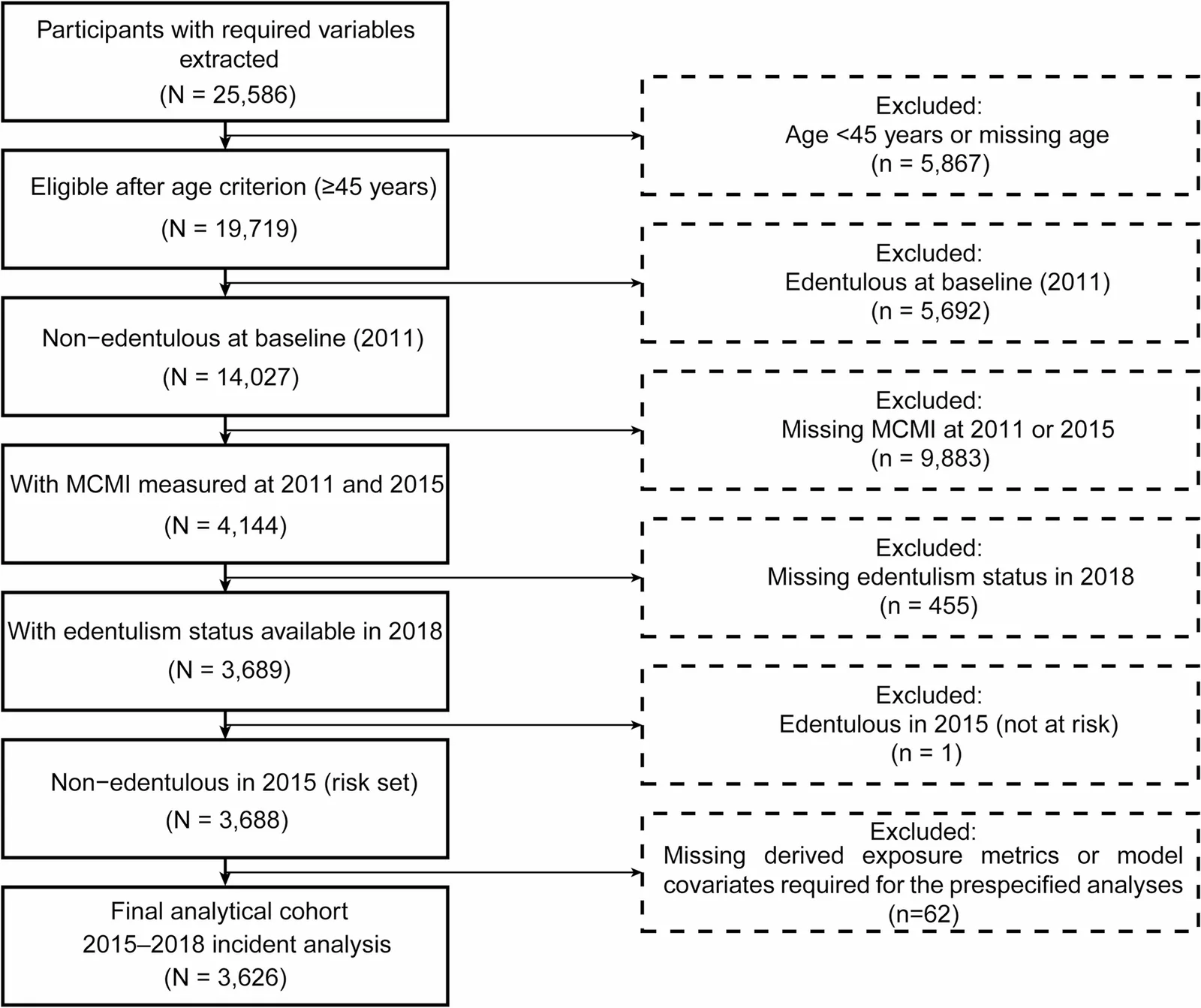
Flow diagram of participant selection for the 2015–2018 incident edentulism analysis in CHARLS

Participant selection is summarized in Fig. 1. In brief, individuals were eligible if they were aged 45 years or older, had the information required to compute MCMI in both 2011 and 2015, and had edentulism status recorded in 2018. To focus on incident edentulism, the risk set was restricted to participants who were not edentulous in 2011 and remained non edentulous in 2015, so all participants contributed risk time starting from 2015. After derivation of exposure metrics and assembly of the prespecified covariate set, we excluded participants who could not be included in the planned regression models because required variables for exposure construction, outcome ascertainment, or model specification were unavailable (Fig. 1). For covariates with missingness, we retained participants by modeling missingness as a separate category, consistent with Table 1; the full baseline profile including additional health and psychosocial variables is provided in Table S2. MCMI-related exposure metrics and province-level coverage by MCMI change-pattern group are summarized in Table S3. Missingness across variables used to construct MCMI-based exposures, ascertain incident edentulism, and describe baseline characteristics is summarized in Table S1. The final analytical cohort comprised 3,626 participants.

### Exposure assessment: modified cardiometabolic index (MCMI)

MCMI was calculated in 2011 and 2015 using fasting biomarkers and anthropometry with the following formula [15, 25].

$$\mathrm{MCMI}=\ln\left[\frac{\mathrm{TG}\times\mathrm{FBG}}{\mathrm{HDL-C}}\right]\times\left(\frac{\mathrm{WC}}{\mathrm{height}}\right)$$

where TG is triglycerides, FBG is fasting blood glucose, and HDL-C is high-density lipoprotein cholesterol. TG, FBG, and HDL-C were converted to mg/dL using standard conversion factors prior to index derivation. WC and height were expressed in cm, and ln denotes the natural logarithm.

MCMI was treated as an integrated summary of cardiometabolic burden. It combines glycemic, lipid, and central adiposity components within a single index, which allowed us to characterize cardiometabolic status across the 2011–2015 exposure window. Although MCMI has been used as a cardiometabolic risk marker in previous studies, it has not been validated as an edentulism-specific prediction tool. We therefore used it to summarize cardiometabolic exposure rather than to define a diagnostic or clinical prediction category for complete tooth loss.

Because cardiometabolic burden may matter through both its overall level and how it changes over time, MCMI was represented in three complementary ways. First, participants were assigned to one of four two-wave MCMI change patterns that reflected stability and direction of change between 2011 and 2015. High versus low MCMI at each wave was defined using the wave-specific median, yielding stable high, high-to-low, low-to-high, and stable low change patterns. Because no clinically established MCMI cutoff exists for edentulism risk, wave-specific medians were used to define distribution-based high and low MCMI at each assessment. This approach was intended to summarize starting level and direction of change without imposing unsupported clinical thresholds. Second, change was quantified as ΔMCMI = MCMI_2015 − MCMI_2011 and categorized into tertiles. Third, cumulative exposure was approximated as the average of the two measurements multiplied by the 4-year interval, cumulative MCMI = [(MCMI_2011 + MCMI_2015)/2] × 4, and similarly divided into tertiles, with a trend test treating tertiles as an ordinal term. Tertile analyses were retained as descriptive categorical summaries and were not interpreted as evidence that the underlying associations were threshold-based.

**Fig. 2:**
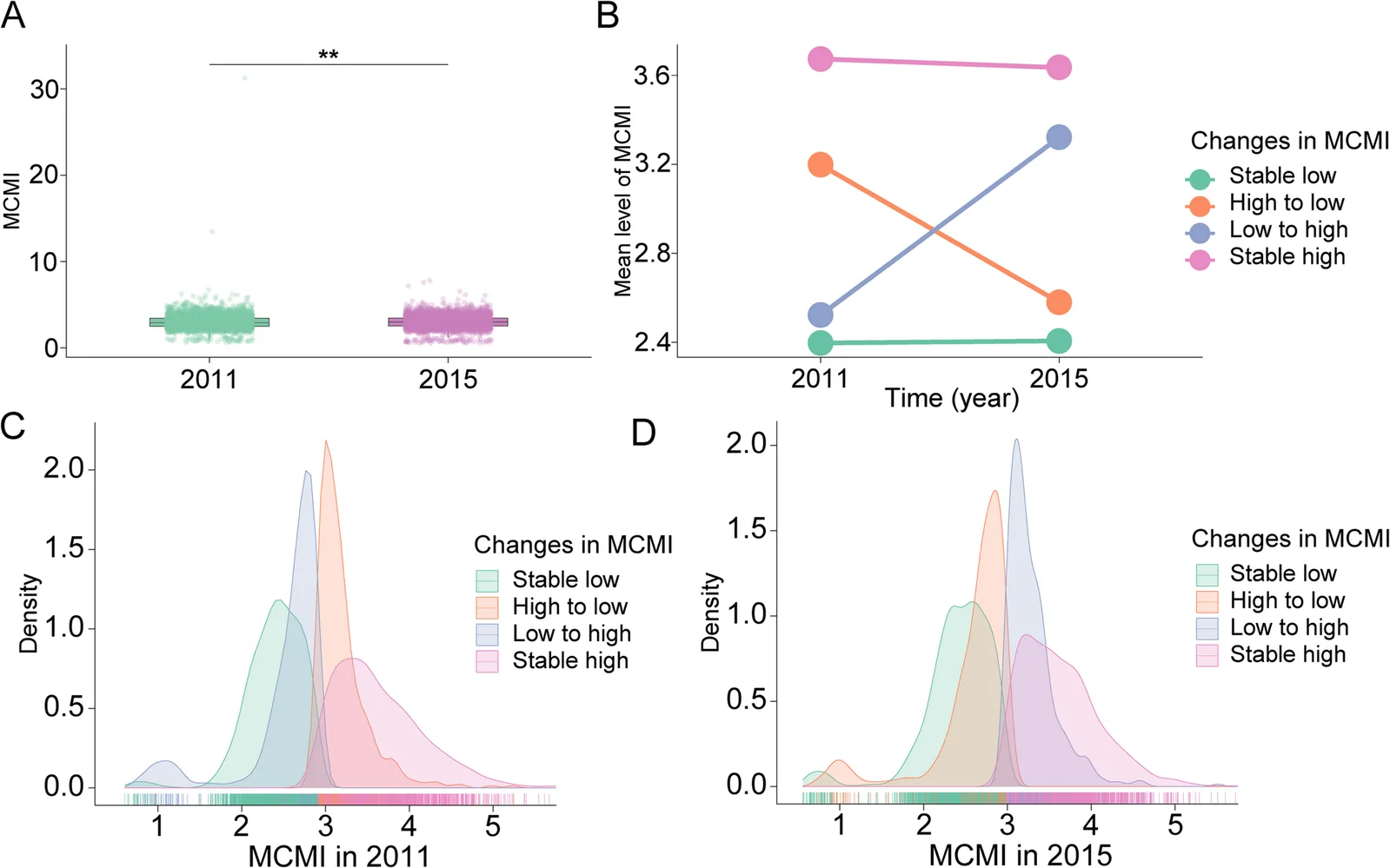
Distribution and two-wave change patterns of the modified cardiometabolic index between 2011 and 2015 in the analytical cohort. **A **Individual paired MCMI values in 2011 and 2015. **B** Mean MCMI at each wave by two-wave MCMI change pattern (stable low, high to low, low to high, stable high). **C **Density distributions of MCMI in 2011 within each two-wave MCMI change pattern. **D **Density distributions of MCMI in 2015 within each two-wave MCMI change pattern

Figure 2 provides a visual summary of MCMI across the exposure window. It contrasts paired MCMI values in 2011 and 2015 (Fig. 2A), shows the group-level profiles over time (Fig. 2B), and displays the distribution of MCMI at each wave within each two-wave change pattern (Fig. 2C-D).

### Outcome definition and follow-up

The outcome was incident edentulism from 2015 to 2018, based on the CHARLS item asking whether the respondent had lost all teeth (yes/no). This item is the standard CHARLS indicator for edentulism and aligns well with population-level surveillance of complete tooth loss. Participants were considered at risk if they were non-edentulous in 2015 and had edentulism status recorded in 2018. Incident edentulism was defined as reporting complete tooth loss in 2018 among those at risk in 2015.

### Covariates

All covariates used for regression adjustment were assessed at baseline (Wave 1, 2011), unless otherwise stated. For the primary regression models, we adjusted for age group, sex, geographic region (East, Central, West), smoking status, drinking status, and body mass index category. We further adjusted for education level, standard of living, and sleep duration. To interpret MCMI change patterns in the context of starting cardiometabolic status, Model 3 additionally included baseline MCMI.

Clinical conditions reported in CHARLS were summarised descriptively at baseline by MCMI change-pattern group but were not included in the primary models. Several of these conditions may lie on the pathway linking cardiometabolic dysregulation to tooth loss, and some overlap conceptually with the cardiometabolic construct captured by MCMI, which increases the risk of overadjustment and multicollinearity.

Province was presented descriptively to show geographic coverage but was not included in multivariable models because of sparse counts across many categories, and because the broader region indicator already captured large scale geographic variation.

### Statistical analysis

Baseline characteristics were summarized overall and by two-wave MCMI change pattern. Continuous variables are reported as median (interquartile range), and categorical variables as n (percent). Between-group differences were assessed using the Kruskal–Wallis test for continuous variables and the chi-square test for categorical variables.

Associations with incident edentulism were estimated as risk ratios with 95% confidence intervals using Poisson regression with robust variance [26, 27]. The stable high change pattern served as the reference group in the primary analysis. Adjustment was introduced stepwise. Model 1 included age, sex, geographic region, smoking status, drinking status, and body mass index category. Model 2 additionally included education level, standard of living, and sleep duration. Model 3 further adjusted for baseline MCMI to evaluate two-wave change patterns conditional on starting cardiometabolic status. Accordingly, Model 3 estimates were interpreted as contrasts between change patterns at a similar baseline MCMI level, rather than as unconditioned between-group differences.

Cumulative MCMI and absolute change in MCMI were examined as alternative categorical exposure summaries. For cumulative MCMI, the lowest tertile served as the reference group. For absolute change in MCMI, ΔMCMI was categorized into tertiles, with T3 used as the reference category to match the prespecified presentation. Ordinal trend tests were performed by modeling tertile categories as ordered terms. To address potential information loss from categorization, we additionally examined MCMI in 2011, MCMI in 2015, ΔMCMI, and cumulative MCMI as continuous variables scaled per standard deviation. Spline-based analyses using the Model 2 covariate structure were used to visualize potential dose-response patterns, with risk ratios referenced to the median value of each exposure.

For missing covariate data, the primary models retained missing values as explicit categories for categorical covariates to preserve the prespecified analytic cohort. Because this approach may be sensitive to informative missingness, we performed a multiple-imputation sensitivity analysis for covariates with missing values. Exposure variables and incident edentulism were not imputed. The imputation model included the outcome, exposure group, and all covariates used in the analytic models, and the primary two-wave MCMI change-pattern analysis was repeated across imputed datasets [28].

To examine potential selection into the final analytic cohort, we compared baseline characteristics between participants included in the final analysis and those excluded after the baseline non-edentulous risk set, using absolute standardized mean differences. Subgroup analyses repeated Model 3 within strata of sex and age group and were interpreted as exploratory. Logistic regression was used as a sensitivity analysis to assess whether the direction of association was consistent under a conventional binary-outcome modeling framework; modified Poisson regression with robust variance was retained as the primary approach because it directly estimates risk ratios. All analyses were conducted in R version 4.5.1. Tests were two-sided, and P values below 0.05 were considered statistically significant.

## Results

### Study sample and analytical pipeline

Drawing from CHARLS, we identified 25,586 participants at the 2011 baseline (Fig. 1). After restricting to adults aged 45 years or older (*n* = 19,719) and excluding those with baseline edentulism (*n* = 5,692), 14,027 remained at risk. We then required MCMI to be available in both 2011 and 2015 (*n* = 4,144) and edentulism status to be ascertainable in 2018 (*n* = 3,689). After excluding one participant who was edentulous in 2015 and therefore not at risk for incident edentulism during 2015 to 2018, as well as 62 participants who could not be included in the prespecified models after exposure derivation and covariate assembly (Fig. 1), the final analytical cohort comprised 3,626 individuals.

Missingness among covariates used for regression adjustment was primarily observed in self reported variables, while the exposure metrics and outcome ascertainment variables were complete in the final cohort (Table S1). Baseline characteristics are presented overall and by two-wave MCMI change pattern group in Table 1, with the full baseline covariate profile and *P* values provided in Table S2. MCMI-related exposure metrics and province-level coverage are summarized in Table S3.

Wave 1 (2011) provided baseline information and the first MCMI assessment, Wave 3 (2015) provided the second MCMI assessment and defined the risk set of participants who were non-edentulous in 2015, and Wave 4 (2018) ascertained incident edentulism during follow-up.

### Baseline profile and MCMI evolution

Baseline characteristics are summarized in Table 1, with expanded baseline covariates and P values provided in Table S2. At baseline, 31.2% of participants were aged ≥ 65 years, 43.3% were aged 55–64 years 55–65 years, and 25.5% were aged 45–54 years 45–55 years; 56.3% were female. Cardiometabolic burden was common (hypertension 36.3%, diabetes 10.5%, dyslipidaemia 21.6%), while behavioral risk gradients were substantial (ever smoking 41.2%, alcohol drinking 33.2%). Notably, cardiometabolic and lifestyle profiles did not move in lockstep: participants with lower cardiometabolic burden tended to report higher smoking and drinking, whereas those with higher cardiometabolic burden showed greater adiposity and a higher prevalence of diagnosed conditions.

**Table 1:** Baseline characteristics by two-wave MCMI change pattern group in the analytical cohort (CHARLS 2011–2018)

Characteristic	Overall (*n* = 3626)	Two-wave MCMI change pattern group				
		Stable high (*n* = 1408)	High to low (*n* = 405)	Low to high (*n* = 405)	Stable low (*n* = 1408)	*P* value
Age						< 0.001
≥65 years	1130 (31.2%)	436 (31.0%)	132 (32.6%)	91 (22.5%)	471 (33.5%)	
45–54 years	925 (25.5%)	352 (25.0%)	83 (20.5%)	129 (31.9%)	361 (25.6%)	
55–64 years	1571 (43.3%)	620 (44.0%)	190 (46.9%)	185 (45.7%)	576 (40.9%)	
Sex						< 0.001
Female	2040 (56.3%)	936 (66.5%)	214 (52.8%)	257 (63.5%)	633 (45.0%)	
Male	1586 (43.7%)	472 (33.5%)	191 (47.2%)	148 (36.5%)	775 (55.0%)	
Region						< 0.001
Central	1442 (39.8%)	596 (42.3%)	156 (38.5%)	135 (33.3%)	555 (39.4%)	
East	1185 (32.7%)	491 (34.9%)	121 (29.9%)	161 (39.8%)	412 (29.3%)	
Missing	4 (0.1%)	4 (0.3%)	0 (0.0%)	0 (0.0%)	0 (0.0%)	
West	995 (27.4%)	317 (22.5%)	128 (31.6%)	109 (26.9%)	441 (31.3%)	
Education level						0.194
Elementary school	809 (22.3%)	304 (21.6%)	96 (23.7%)	88 (21.7%)	321 (22.8%)	
High school or above	384 (10.6%)	135 (9.6%)	49 (12.1%)	52 (12.8%)	148 (10.5%)	
Less than elementary school	1649 (45.5%)	668 (47.4%)	166 (41.0%)	166 (41.0%)	649 (46.1%)	
Middle school	784 (21.6%)	301 (21.4%)	94 (23.2%)	99 (24.4%)	290 (20.6%)	
Standard of living						0.196
Average	1272 (35.1%)	501 (35.6%)	149 (36.8%)	153 (37.8%)	469 (33.3%)	
Missing	1497 (41.3%)	561 (39.8%)	155 (38.3%)	176 (43.5%)	605 (43.0%)	
Poor	279 (7.7%)	112 (8.0%)	35 (8.6%)	22 (5.4%)	110 (7.8%)	
Relatively high	102 (2.8%)	42 (3.0%)	11 (2.7%)	17 (4.2%)	32 (2.3%)	
Relatively poor	455 (12.5%)	182 (12.9%)	53 (13.1%)	36 (8.9%)	184 (13.1%)	
Very high	21 (0.6%)	10 (0.7%)	2 (0.5%)	1 (0.2%)	8 (0.6%)	
Sleep duration						0.545
≤6 h	334 (9.2%)	137 (9.7%)	41 (10.1%)	36 (8.9%)	120 (8.5%)	
6-8h	1846 (50.9%)	687 (48.8%)	210 (51.9%)	208 (51.4%)	741 (52.6%)	
8 h	1363 (37.6%)	545 (38.7%)	147 (36.3%)	155 (38.3%)	516 (36.6%)	
Missing	83 (2.3%)	39 (2.8%)	7 (1.7%)	6 (1.5%)	31 (2.2%)	
Smoking Status						< 0.001
No	2133 (58.8%)	924 (65.6%)	232 (57.3%)	265 (65.4%)	712 (50.6%)	
Yes	1493 (41.2%)	484 (34.4%)	173 (42.7%)	140 (34.6%)	696 (49.4%)	
Drinking Status						< 0.001
Missing	13 (0.4%)	4 (0.3%)	1 (0.2%)	2 (0.5%)	6 (0.4%)	
No	2409 (66.4%)	1032 (73.3%)	268 (66.2%)	289 (71.4%)	820 (58.2%)	
Yes	1204 (33.2%)	372 (26.4%)	136 (33.6%)	114 (28.1%)	582 (41.3%)	
Body mass index category						< 0.001
Underweight	160 (4.4%)	4 (0.3%)	11 (2.7%)	1 (0.2%)	144 (10.2%)	
Normal weight	1675 (46.2%)	272 (19.3%)	247 (61.0%)	139 (34.3%)	1017 (72.2%)	
Overweight	1265 (34.9%)	691 (49.1%)	131 (32.3%)	214 (52.8%)	229 (16.3%)	
Obese	513 (14.1%)	437 (31.0%)	16 (4.0%)	47 (11.6%)	13 (0.9%)	
Missing	13 (0.4%)	4 (0.3%)	0 (0.0%)	4 (1.0%)	5 (0.4%)	
Hypertension						< 0.001
Missing	67 (1.8%)	33 (2.3%)	8 (2.0%)	6 (1.5%)	20 (1.4%)	
No	2244 (61.9%)	692 (49.1%)	245 (60.5%)	249 (61.5%)	1058 (75.1%)	
Yes	1315 (36.3%)	683 (48.5%)	152 (37.5%)	150 (37.0%)	330 (23.4%)	
Dyslipidaemia						< 0.001
Missing	143 (3.9%)	64 (4.5%)	22 (5.4%)	11 (2.7%)	46 (3.3%)	
No	2698 (74.4%)	898 (63.8%)	303 (74.8%)	299 (73.8%)	1198 (85.1%)	
Yes	785 (21.6%)	446 (31.7%)	80 (19.8%)	95 (23.5%)	164 (11.6%)	
Diabetes						< 0.001
Missing	94 (2.6%)	44 (3.1%)	13 (3.2%)	8 (2.0%)	29 (2.1%)	
No	3150 (86.9%)	1113 (79.0%)	352 (86.9%)	368 (90.9%)	1317 (93.5%)	
Yes	382 (10.5%)	251 (17.8%)	40 (9.9%)	29 (7.2%)	62 (4.4%)	

Values are presented as *n* (%) for categorical variables. Missing values for covariates were retained as a separate category in descriptive summaries and in regression models, as prespecified. *P* values compare characteristics across two-wave MCMI change pattern groups using the chi-square test

Across the cohort, MCMI changed only modestly between waves (median 2.91 in 2011 to 2.98 in 2015; Table S3). The two-wave classification identified four change patterns, visualized in Fig. 2 and summarized in Table S3: stable high, high-to-low, low-to-high, and stable low. In the stable high group, MCMI remained consistently elevated (median 3.53 to 3.56). The high-to-low pattern showed a marked decline (3.11 to 2.72; median ΔMCMI − 0.44), whereas the low-to-high pattern shifted upward (2.70 to 3.20; median ΔMCMI + 0.56). The stable low group remained persistently low (2.43 to 2.47). These patterns provided the framework for the primary analysis of incident edentulism.

### Incident edentulism by two-wave MCMI change pattern

During follow-up from 2015 to 2018, 231 participants developed edentulism, yielding a 6.4% cumulative incidence (Table 2). Incidence varied across two-wave MCMI change patterns. The high-to-low pattern had the lowest incidence (4.2%, 17/405), stable high was intermediate (6.1%, 86/1,408), the low-to-high pattern was 5.2% (21/405), and stable low was highest (7.6%, 107/1,408) (Table 2).

Crude incidence differed across the four two-wave MCMI change patterns and was therefore followed by adjusted regression analyses. The stable low group showed the highest crude incidence, whereas the high-to-low group showed the lowest crude incidence. Because baseline characteristics differed across MCMI change-pattern groups, adjusted models were used to estimate associations while accounting for prespecified sociodemographic, behavioral, and anthropometric covariates (Table 1).

### Primary association analysis

We estimated risk ratios (RRs) using Poisson regression with robust variance, with stable high as the reference group. After adjustment, stable low did not show evidence of increased risk despite its higher crude incidence. In Model 3, the RR for stable low was 0.90 (95% CI 0.60 to 1.36; *P* = 0.627), with similarly null estimates in Models 1 and 2 (Table 2). This pattern indicates that the crude excess observed in the stable low group was not sustained once key covariates were accounted for.

**Table 2:** Associations of two-wave MCMI change patterns with incident edentulism

		Model 1		Model 2		Model 3	
	Event (%)	RR (95% CI)	*P* value	RR (95% CI)	*P* value	RR (95% CI)	*P* value
Stable high	86 (6.1%)	Reference		Reference		Reference	
High to low	17 (4.2%)	0.57 (0.34–0.97)	0.037	0.59 (0.35–1.00)	0.05	0.57 (0.34–0.97)	0.037
Low to high	21 (5.2%)	0.87 (0.55–1.40)	0.572	0.89 (0.56–1.42)	0.63	0.81 (0.48–1.37)	0.432
Stable low	107 (7.6%)	0.98 (0.69–1.39)	0.922	1.00 (0.71–1.42)	0.997	0.90 (0.60–1.36)	0.627

Model 1 was adjusted for age, sex, geographic region (East, Central, West), smoking status, drinking status, and body mass index category. Model 2 was further adjusted for education level, standard of living, and sleep duration. Model 3 was additionally adjusted for baseline modified cardiometabolic index (MCMI). Risk ratios (RRs) and 95% confidence intervals (CIs) were estimated using Poisson regression with robust variance, with the stable high change pattern as the reference group. Because follow-up was uniformly from 2015 to 2018 for all participants, RRs can be interpreted as cumulative risk ratios over this period

The high-to-low pattern was associated with a lower risk of incident edentulism across models. The RR was 0.57 (95% CI 0.34 to 0.97; *P* = 0.037) in Model 1, 0.59 (95% CI 0.35 to 1.00; *P* = 0.050) in Model 2, and 0.57 (95% CI 0.34 to 0.97; *P* = 0.037) in Model 3 (Table 2). This estimate was directionally consistent with the logistic regression sensitivity analysis (Table S5). As a descriptive comparison, the observed incidence in the high-to-low group (4.2%) was 1.9% points lower than in the stable high group (6.1%). The low-to-high pattern showed no clear association after full adjustment (RR 0.81, 95% CI 0.48 to 1.37; *P* = 0.432) (Table 2). Overall, the primary Poisson models and logistic regression sensitivity analysis yielded directionally consistent estimates for the high-to-low two-wave MCMI change pattern (Table 2; Table S5).

### Associations for cumulative MCMI and absolute MCMI change

Associations for cumulative MCMI are shown in Table 3. In the overall cohort, cumulative MCMI tertiles did not show a consistent graded association with incident edentulism. In Model 3, the RR was 0.90 (95% CI 0.62 to 1.32; *P* = 0.596) for T2 and 1.65 (95% CI 0.98 to 2.79; *P* = 0.061) for T3 compared with T1, and the ordinal trend was not statistically significant (P for trend = 0.142). Results stratified by baseline MCMI also did not show a consistent monotonic pattern (Table 3).

**Table 3:** Associations of cumulative modified cardiometabolic index with incident edentulism

		Model 1		Model 2		Model 3	
	Event (%)	RR (95% CI)	*P* value	RR (95% CI)	*P* value	RR (95% CI)	*P* value
T1 of cumulative MCMI	91 (7.5%)	Reference		Reference		Reference	
T2 of cumulative MCMI	61 (5.0%)	0.77 (0.55–1.07)	0.121	0.77 (0.55–1.08)	0.131	0.90 (0.62–1.32)	0.596
T3 of cumulative MCMI	79 (6.5%)	1.17 (0.80–1.70)	0.429	1.15 (0.78–1.68)	0.486	1.65 (0.98–2.79)	0.061
Per-tertile increase (ordinal)	231 (6.4%)	1.06 (0.86–1.30)	0.588	1.05 (0.86–1.29)	0.636	1.23 (0.93–1.63)	0.142

Model 1 was adjusted for age, sex, geographic region (East, Central, West), smoking status, drinking status, and body mass index category; Model 2 was further adjusted for education level, standard of living, and sleep duration; Model 3 was additionally adjusted for baseline modified cardiometabolic index (MCMI). *P* for trend was obtained by modeling tertile category as an ordinal variable (1–3)

A similar pattern was observed for absolute change in MCMI (ΔMCMI). When ΔMCMI was categorized into tertiles with T3 as the reference, associations were close to the null in the overall cohort. In Model 3, the RR was 1.09 (95% CI 0.77 to 1.54; *P* = 0.619) for T1 and 1.04 (95% CI 0.75 to 1.43; *P* = 0.826) for T2, with no evidence of an ordinal trend (P for trend = 0.618) (Table 4). After stratification by baseline MCMI, estimates remained non-graded and imprecise. Among participants with high baseline MCMI, T2 showed a nonsignificant elevation relative to T3 (RR 1.64, 95% CI 0.94 to 2.88; *P* = 0.081), while the trend test remained non-significant (P for trend = 0.613). Estimates in the low baseline MCMI stratum were close to the null, with no evidence of trend (P for trend = 0.731) (Table 4). Overall, cumulative MCMI and ΔMCMI tertiles did not show consistent graded associations with incident edentulism in these categorical analyses.

**Table 4:** Associations of tertiles of absolute change in MCMI with incident edentulism

		Model 1		Model 2		Model 3	
	Event (%)	RR (95% CI)	*P* value	RR (95% CI)	*P* value	RR (95% CI)	*P* value
All participants							
T3 of ΔMCMI	71 (5.9%)	Reference		Reference		Reference	
T1 of ΔMCMI	83 (6.9%)	1.02 (0.75–1.38)	0.921	1.01 (0.74–1.38)	0.932	1.09 (0.77–1.54)	0.619
T2 of ΔMCMI	77 (6.4%)	1.00 (0.73–1.38)	0.995	1.01 (0.73–1.38)	0.972	1.04 (0.75–1.43)	0.826
Trend test	231 (6.4%)	0.99 (0.85–1.16)	0.919	0.99 (0.85–1.16)	0.932	0.96 (0.80–1.14)	0.618
Participants with high baseline MCMI							
T3 of ΔMCMI	18 (4.2%)	Reference		Reference		Reference	
T1 of ΔMCMI	47 (5.7%)	1.25 (0.72–2.15)	0.424	1.23 (0.71–2.13)	0.456	1.26 (0.71–2.22)	0.427
T2 of ΔMCMI	38 (6.8%)	1.64 (0.94–2.86)	0.079	1.64 (0.94–2.86)	0.083	1.64 (0.94–2.88)	0.081
Trend test	103 (5.7%)	0.95 (0.75–1.20)	0.66	0.96 (0.76–1.21)	0.709	0.94 (0.73–1.20)	0.613
Participants with low baseline MCMI							
T3 of ΔMCMI	53 (6.8%)	Reference		Reference		Reference	
T1 of ΔMCMI	36 (9.5%)	1.11 (0.73–1.68)	0.64	1.10 (0.72–1.70)	0.65	1.13 (0.71–1.79)	0.613
T2 of ΔMCMI	39 (6.0%)	0.76 (0.50–1.15)	0.198	0.78 (0.51–1.19)	0.247	0.79 (0.52–1.21)	0.273
Per-tertile increase (ordinal)	128 (7.1%)	0.97 (0.77–1.23)	0.801	0.97 (0.77–1.22)	0.79	0.96 (0.74–1.23)	0.731

ΔMCMI was calculated as the modified cardiometabolic index at Wave 3 minus that at baseline (Wave 1). Participants were categorized into tertiles according to the distribution of ΔMCMI, with T1 representing the lowest tertile, T2 the middle tertile, and T3 the highest tertile. T3 of ΔMCMI was used as the reference category. Participants were further stratified into high and low baseline MCMI groups according to the median value of baseline MCMI. Model 1 was adjusted for age, sex, geographic region (East, Central, West), smoking status, drinking status, and body mass index category; Model 2 was further adjusted for education level, standard of living, and sleep duration; Model 3 was additionally adjusted for baseline modified cardiometabolic index (MCMI)

### Additional and sensitivity analyses

Age-stratified analyses suggested potential heterogeneity by age group (Table S4). The low-to-high pattern was associated with a lower risk among participants aged ≥ 65 years (RR 0.25, 95% CI 0.09 to 0.70; *P* = 0.008), whereas an elevated risk estimate was observed among those aged 45 to 55 years (RR 3.20, 95% CI 1.21 to 8.46; *P* = 0.019). Because these stratum-specific estimates were based on smaller numbers of events and yielded wide confidence intervals, they should be interpreted cautiously and regarded as hypothesis-generating, and we did not perform formal tests of interaction in the present analyses. Sex-stratified associations were broadly similar (Table S4).

In logistic regression sensitivity analyses, the high-to-low pattern remained associated with lower odds of incident edentulism after full adjustment (OR 0.56, 95% CI 0.31 to 1.00; *P* = 0.049), while the stable low and low-to-high patterns showed no clear association (Table S5). Overall, the logistic regression sensitivity analysis was directionally consistent with the primary Poisson models, whereas subgroup estimates were imprecise and were treated as exploratory.

Continuous exposure analyses provided no evidence of a consistent association between MCMI summaries and incident edentulism (Table S6). In Model 2, the RRs per 1-SD increase were 0.93 (95% CI 0.79 to 1.10; *P* = 0.422) for MCMI in 2011, 1.00 (95% CI 0.86 to 1.17; *P* = 0.969) for MCMI in 2015, 1.07 (95% CI 0.94 to 1.21; *P* = 0.325) for ΔMCMI, and 0.96 (95% CI 0.81 to 1.13; *P* = 0.624) for cumulative MCMI. Spline analyses were consistent with these findings and did not show clear dose-response patterns (Figure S1).

The multiple-imputation sensitivity analysis yielded results similar to the primary complete-data analysis (Table S7). Compared with the stable high pattern, the high-to-low pattern remained associated with a lower risk of incident edentulism (RR 0.57, 95% CI 0.34 to 0.96; *P* = 0.036), whereas the low-to-high pattern (RR 0.79, 95% CI 0.47 to 1.33; *P* = 0.378) and stable low pattern (RR 0.91, 95% CI 0.60 to 1.36; *P* = 0.631) showed no clear associations.

Included-versus-excluded comparisons showed that participants in the final analytic cohort differed from those excluded after the baseline non-edentulous risk set on several baseline characteristics (Table S8). The largest imbalances were observed for standard of living, stroke, age, diabetes, heart disease, and hypertension, supporting the need to interpret the findings in light of potential selection into the final analytic cohort.

## Discussion

In this longitudinal CHARLS analysis of 3,626 middle-aged and older Chinese adults with natural teeth in 2015, 231 participants became edentulous by 2018. Compared with the stable high MCMI pattern, the high-to-low pattern was associated with a lower risk of incident edentulism, and this estimate remained similar after adjustment for baseline MCMI and in the multiple-imputation sensitivity analysis. The stable low group had the highest crude incidence, but this excess was not sustained after covariate adjustment. Cumulative MCMI tertiles, ΔMCMI tertiles, continuous MCMI summaries, and spline analyses did not show consistent graded or dose-response associations. These findings indicate an association between two-wave cardiometabolic change patterns and subsequent edentulism, while also emphasizing the need for cautious interpretation because complete tooth loss is a late-stage cumulative oral health endpoint.

The observed pattern should be interpreted within the broader epidemiology of tooth loss. Edentulism reflects cumulative exposure to oral disease, health behaviors, access to preventive and restorative dental care, and treatment decisions over many years. National oral health survey data from China have shown substantial tooth loss and incomplete prosthetic rehabilitation among adults, with geographic disparities in restoration patterns [3]. Oral diseases are also strongly shaped by social conditions, commercial exposures, and health-system access, which can influence both prevention and late-stage outcomes such as complete tooth loss [29]. The attenuation of the stable low group’s higher crude incidence after adjustment is consistent with this broader context: differences in smoking, alcohol use, adiposity, socioeconomic indicators, and geographic context may create crude oral health gradients that are not explained by cardiometabolic biomarkers alone.

Several biological pathways make cardiometabolic–oral health associations plausible, although they cannot establish causality in the present study. Metabolic syndrome components, including central adiposity, dyslipidemia, hyperglycemia, and elevated blood pressure, are linked to inflammatory and vascular processes relevant to periodontal tissue homeostasis [8, 10]. Insulin resistance has also been associated with periodontal inflammation, with vascular factors such as blood pressure potentially contributing to this relationship [12]. Periodontitis and diabetes have a well-recognized bidirectional relationship, and periodontal inflammation may contribute to systemic inflammatory burden [30]. Vascular endothelial dysfunction provides another plausible bridge between periodontal inflammation and systemic cardiometabolic disease [31]. These mechanisms support the biological relevance of studying cardiometabolic burden in relation to tooth loss, but they do not imply that a short-term change in MCMI directly caused or prevented complete tooth loss.

The oral endpoint is especially important for interpretation. Periodontitis is a chronic inflammatory disease whose staging reflects severity, complexity, and progression over time [32]. Complete tooth loss is further downstream, because it can arise from periodontal destruction, severe caries, extraction decisions, dental attendance patterns, prosthetic rehabilitation pathways, and accumulated social disadvantage. Periodontitis also occurs within multimorbidity contexts involving diabetes and obesity, reinforcing the need to view oral and metabolic conditions as interconnected rather than isolated disease processes [33]. Therefore, the high-to-low MCMI association may reflect a broader systemic, behavioral, or care-related risk profile rather than a single cardiometabolic pathway leading directly to edentulism.

Our findings also fit within prior epidemiologic evidence, while addressing a different aspect of the question. Composite cardiometabolic indices such as CMI and MCMI have been used as integrated summaries of metabolic risk and have been associated with outcomes such as type 2 diabetes, non-alcoholic fatty liver disease, liver fibrosis, and cardiovascular events [13–16, 25]. In oral health research, higher CMI has been associated with periodontitis in US adults [17], and a systematic review and meta-analysis found that metabolic syndrome was associated with tooth loss [18]. Studies from Japan and South Korea have also reported associations between metabolic syndrome and tooth loss burden [19, 20]. In CHARLS, lipid profiles have been linked to reported edentulism, supporting the relevance of metabolic–oral health questions in Chinese ageing cohorts [21]. Our study extends this literature by focusing on incident edentulism after a defined two-wave cardiometabolic exposure window, while recognizing that two MCMI measurements cannot characterize long-term metabolic history.

The alternative exposure analyses help define the boundary of the main finding. Cumulative MCMI and ΔMCMI tertiles did not show consistent monotonic associations, and continuous MCMI models and spline analyses did not show clear dose-response patterns. These results argue against a simple interpretation in which higher cumulative cardiometabolic burden or greater absolute change alone explains incident edentulism. The two-wave change-pattern categories summarized starting level and direction of change during the exposure window, but they should not be interpreted as a true trajectory model or as inherently superior to continuous or cumulative summaries. Differences across exposure specifications may reflect the measurement window, categorization choices, event numbers, and the late-stage nature of edentulism as much as underlying biology.

The sensitivity analyses were broadly consistent with the main analysis while clarifying its limitations. Logistic regression yielded estimates directionally consistent with the modified Poisson models; the latter was retained as the primary approach because it directly estimates risk ratios for cohort outcomes [26, 27]. The multiple-imputation sensitivity analysis also yielded results similar to the primary analysis, reducing concern that the missing-category approach alone explained the observed association [28]. However, the included-versus-excluded comparison showed measurable differences between participants retained in the final analytic cohort and those excluded after the baseline non-edentulous risk set. Selection into the analytic sample may therefore affect generalizability and should be considered when interpreting the results. The age-stratified estimates were imprecise and based on smaller event numbers, so they are best viewed as exploratory.

This study has several limitations. First, edentulism was self-reported, and the data did not capture the clinical pathways leading to complete tooth loss. We could not distinguish teeth lost because of periodontal disease, caries, trauma, access barriers, or treatment decisions. Second, baseline oral health information was limited. Although we excluded participants who were already edentulous before the outcome window, we could not adjust for number of remaining teeth, periodontal status, caries history, tooth mobility, oral hygiene, dental attendance, denture use, or prosthodontic access. Third, MCMI was measured at only two time points, so the exposure categories should be interpreted as two-wave change patterns rather than long-term trajectories. Fourth, wave-specific median cut points and tertile-based summaries were distribution-based and may not correspond to clinically meaningful thresholds for edentulism risk. Fifth, missing covariate data and the large reduction from the baseline non-edentulous risk set to the final analytic cohort may have introduced selection bias despite sensitivity analyses. Finally, residual confounding remains possible because diet quality, fluoride exposure, dental-care access, oral hygiene behaviors, and socioeconomic conditions were incompletely measured.

These findings have implications for oral epidemiology and ageing research. They do not establish that cardiometabolic improvement prevents complete tooth loss, and they should not be used as evidence for cardiometabolic intervention as an isolated oral health strategy. A more appropriate interpretation is that cardiometabolic profiles may help characterize broader risk contexts in which late-stage oral health loss occurs. This interpretation is consistent with the common risk factor approach, which places oral health within broader social, behavioral, and chronic disease frameworks [34]. It also aligns with evidence linking oral health and systemic noncommunicable diseases across multiple conditions [35]. Future studies with repeated cardiometabolic measurements, detailed baseline oral examinations, tooth-level outcomes, dental service information, and competing-risk or time-varying frameworks will be better positioned to clarify whether cardiometabolic change contributes independently to tooth-loss progression or primarily marks shared systemic, behavioral, and access-related vulnerability [36].

## Conclusion

In this longitudinal CHARLS analysis, a high-to-low MCMI change pattern from 2011 to 2015 was associated with a lower risk of incident edentulism from 2015 to 2018 compared with a stable high pattern. Cumulative MCMI, absolute MCMI change, continuous MCMI summaries, and spline analyses did not show consistent graded associations. Because complete tooth loss is a late-stage cumulative endpoint and detailed baseline oral health measures were unavailable, these findings should be interpreted as observational associations rather than evidence of a direct cardiometabolic effect on tooth loss. Further studies with repeated cardiometabolic assessments and richer oral phenotyping are needed to clarify how systemic metabolic patterns relate to tooth-loss progression.

## Supplementary Information

Supplementary Material 1.

## Data Availability

The data that support the findings of this study are openly available from the China Health and Retirement Longitudinal Study (CHARLS) at https://charls.pku.edu.cn. Further inquiries can be directed to the corresponding author.
