# Supplementary material for: Two-wave modified cardiometabolic index change patterns and incident edentulism in Chinese adults: a longitudinal study from CHARLS
Source: BMC Oral Health. 2026 Aug 13;26:2244. doi: 10.1186/s12903-026-09177-7 (PMC13757159; doi:10.1186/s12903-026-09177-7)
Supplement: Supplementary file 1 [file 12903_2026_9177_MOESM1_ESM.docx]

**Supplementary Materials:**

**Table S1. Missingness overview of variables used in exposure and outcome construction and baseline description (N = 3,626)**

| Variable | Missing_n | Missing_pct |
| --- | --- | --- |
| Age | 0 | 0.0% |
| Sex | 0 | 0.0% |
| Region | 4 | 0.1% |
| Education level | 0 | 0.0% |
| Standard of living | 1497 | 41.3% |
| Sleep duration | 83 | 2.3% |
| Smoking Status | 0 | 0.0% |
| Drinking Status | 13 | 0.4% |
| BMI category | 13 | 0.4% |
| MCMI (Wave 1, 2011) | 0 | 0.0% |
| MCMI (Wave 3, 2015) | 0 | 0.0% |
| Two-wave MCMI change pattern group | 0 | 0.0% |
| Cumulative MCMI | 0 | 0.0% |
| Cumulative MCMI tertile | 0 | 0.0% |
| ΔMCMI | 0 | 0.0% |
| ΔMCMI tertile | 0 | 0.0% |
| Baseline MCMI group | 0 | 0.0% |
| Edentulism (2015) | 0 | 0.0% |
| Edentulism (2018) | 0 | 0.0% |
| Incident edentulism (2015–2018) | 0 | 0.0% |

Number and percentage of missing values are reported for variables used to construct MCMI based exposures, ascertain incident edentulism, and describe baseline characteristics.

**Table S2. Full baseline characteristics of the analytical cohort (Wave 1, 2011) by two-wave MCMI change pattern group, including *P* values.**

| Characteristic | Overall (n=3626) | Two-wave MCMI change pattern group | | | | |
| --- | --- | --- | --- | --- | --- | --- |
|  |  | Stable high (n=1408) | High to low (n=405) | Low to high (n=405) | Stable low (n=1408) | *P* value |
| Age |  |  |  |  |  | <0.001 |
| >=65 | 1130 (31.2%) | 436 (31.0%) | 132 (32.6%) | 91 (22.5%) | 471 (33.5%) |  |
| 45-55 | 925 (25.5%) | 352 (25.0%) | 83 (20.5%) | 129 (31.9%) | 361 (25.6%) |  |
| 55-65 | 1571 (43.3%) | 620 (44.0%) | 190 (46.9%) | 185 (45.7%) | 576 (40.9%) |  |
| Sex |  |  |  |  |  | <0.001 |
| Female | 2040 (56.3%) | 936 (66.5%) | 214 (52.8%) | 257 (63.5%) | 633 (45.0%) |  |
| Male | 1586 (43.7%) | 472 (33.5%) | 191 (47.2%) | 148 (36.5%) | 775 (55.0%) |  |
| Region |  |  |  |  |  | <0.001 |
| Central | 1442 (39.8%) | 596 (42.3%) | 156 (38.5%) | 135 (33.3%) | 555 (39.4%) |  |
| East | 1185 (32.7%) | 491 (34.9%) | 121 (29.9%) | 161 (39.8%) | 412 (29.3%) |  |
| Missing | 4 (0.1%) | 4 (0.3%) | 0 (0.0%) | 0 (0.0%) | 0 (0.0%) |  |
| West | 995 (27.4%) | 317 (22.5%) | 128 (31.6%) | 109 (26.9%) | 441 (31.3%) |  |
| Education level |  |  |  |  |  | 0.194 |
| Elementary school | 809 (22.3%) | 304 (21.6%) | 96 (23.7%) | 88 (21.7%) | 321 (22.8%) |  |
| High school or above | 384 (10.6%) | 135 (9.6%) | 49 (12.1%) | 52 (12.8%) | 148 (10.5%) |  |
| Less than elementary school | 1649 (45.5%) | 668 (47.4%) | 166 (41.0%) | 166 (41.0%) | 649 (46.1%) |  |
| Middle school | 784 (21.6%) | 301 (21.4%) | 94 (23.2%) | 99 (24.4%) | 290 (20.6%) |  |
| Standard of living |  |  |  |  |  | 0.196 |
| Average | 1272 (35.1%) | 501 (35.6%) | 149 (36.8%) | 153 (37.8%) | 469 (33.3%) |  |
| Missing | 1497 (41.3%) | 561 (39.8%) | 155 (38.3%) | 176 (43.5%) | 605 (43.0%) |  |
| Poor | 279 (7.7%) | 112 (8.0%) | 35 (8.6%) | 22 (5.4%) | 110 (7.8%) |  |
| Relatively high | 102 (2.8%) | 42 (3.0%) | 11 (2.7%) | 17 (4.2%) | 32 (2.3%) |  |
| Relatively poor | 455 (12.5%) | 182 (12.9%) | 53 (13.1%) | 36 (8.9%) | 184 (13.1%) |  |
| Very high | 21 (0.6%) | 10 (0.7%) | 2 (0.5%) | 1 (0.2%) | 8 (0.6%) |  |
| Sleep duration |  |  |  |  |  | 0.545 |
| >8 h | 334 (9.2%) | 137 (9.7%) | 41 (10.1%) | 36 (8.9%) | 120 (8.5%) |  |
| ≤6 h | 1846 (50.9%) | 687 (48.8%) | 210 (51.9%) | 208 (51.4%) | 741 (52.6%) |  |
| 6–8 h | 1363 (37.6%) | 545 (38.7%) | 147 (36.3%) | 155 (38.3%) | 516 (36.6%) |  |
| Missing | 83 (2.3%) | 39 (2.8%) | 7 (1.7%) | 6 (1.5%) | 31 (2.2%) |  |
| Smoking Status |  |  |  |  |  | <0.001 |
| No | 2133 (58.8%) | 924 (65.6%) | 232 (57.3%) | 265 (65.4%) | 712 (50.6%) |  |
| Yes | 1493 (41.2%) | 484 (34.4%) | 173 (42.7%) | 140 (34.6%) | 696 (49.4%) |  |
| Drinking Status |  |  |  |  |  | <0.001 |
| Missing | 13 (0.4%) | 4 (0.3%) | 1 (0.2%) | 2 (0.5%) | 6 (0.4%) |  |
| No | 2409 (66.4%) | 1032 (73.3%) | 268 (66.2%) | 289 (71.4%) | 820 (58.2%) |  |
| Yes | 1204 (33.2%) | 372 (26.4%) | 136 (33.6%) | 114 (28.1%) | 582 (41.3%) |  |
| BMI category clean |  |  |  |  |  | <0.001 |
| Underweight | 160 (4.4%) | 4 (0.3%) | 11 (2.7%) | 1 (0.2%) | 144 (10.2%) |  |
| Normal weight | 1675 (46.2%) | 272 (19.3%) | 247 (61.0%) | 139 (34.3%) | 1017 (72.2%) |  |
| Overweight | 1265 (34.9%) | 691 (49.1%) | 131 (32.3%) | 214 (52.8%) | 229 (16.3%) |  |
| Obese | 513 (14.1%) | 437 (31.0%) | 16 (4.0%) | 47 (11.6%) | 13 (0.9%) |  |
| Missing | 13 (0.4%) | 4 (0.3%) | 0 (0.0%) | 4 (1.0%) | 5 (0.4%) |  |
| Depression category |  |  |  |  |  | 0.083 |
| Missing | 1820 (50.2%) | 730 (51.8%) | 180 (44.4%) | 207 (51.1%) | 703 (49.9%) |  |
| No | 953 (26.3%) | 360 (25.6%) | 128 (31.6%) | 94 (23.2%) | 371 (26.3%) |  |
| Yes | 853 (23.5%) | 318 (22.6%) | 97 (24.0%) | 104 (25.7%) | 334 (23.7%) |  |
| Hypertension |  |  |  |  |  | <0.001 |
| Missing | 67 (1.8%) | 33 (2.3%) | 8 (2.0%) | 6 (1.5%) | 20 (1.4%) |  |
| No | 2244 (61.9%) | 692 (49.1%) | 245 (60.5%) | 249 (61.5%) | 1058 (75.1%) |  |
| Yes | 1315 (36.3%) | 683 (48.5%) | 152 (37.5%) | 150 (37.0%) | 330 (23.4%) |  |
| Dyslipidaemia |  |  |  |  |  | <0.001 |
| Missing | 143 (3.9%) | 64 (4.5%) | 22 (5.4%) | 11 (2.7%) | 46 (3.3%) |  |
| No | 2698 (74.4%) | 898 (63.8%) | 303 (74.8%) | 299 (73.8%) | 1198 (85.1%) |  |
| Yes | 785 (21.6%) | 446 (31.7%) | 80 (19.8%) | 95 (23.5%) | 164 (11.6%) |  |
| Diabetes |  |  |  |  |  | <0.001 |
| Missing | 94 (2.6%) | 44 (3.1%) | 13 (3.2%) | 8 (2.0%) | 29 (2.1%) |  |
| No | 3150 (86.9%) | 1113 (79.0%) | 352 (86.9%) | 368 (90.9%) | 1317 (93.5%) |  |
| Yes | 382 (10.5%) | 251 (17.8%) | 40 (9.9%) | 29 (7.2%) | 62 (4.4%) |  |
| Liver disease |  |  |  |  |  | 0.012 |
| Missing | 63 (1.7%) | 18 (1.3%) | 5 (1.2%) | 2 (0.5%) | 38 (2.7%) |  |
| No | 3314 (91.4%) | 1304 (92.6%) | 367 (90.6%) | 378 (93.3%) | 1265 (89.8%) |  |
| Yes | 249 (6.9%) | 86 (6.1%) | 33 (8.1%) | 25 (6.2%) | 105 (7.5%) |  |
| Heart disease |  |  |  |  |  | <0.001 |
| Missing | 82 (2.3%) | 27 (1.9%) | 11 (2.7%) | 9 (2.2%) | 35 (2.5%) |  |
| No | 2875 (79.3%) | 1049 (74.5%) | 332 (82.0%) | 326 (80.5%) | 1168 (83.0%) |  |
| Yes | 669 (18.5%) | 332 (23.6%) | 62 (15.3%) | 70 (17.3%) | 205 (14.6%) |  |
| Stroke |  |  |  |  |  | 0.185 |
| Missing | 36 (1.0%) | 16 (1.1%) | 5 (1.2%) | 2 (0.5%) | 13 (0.9%) |  |
| No | 3453 (95.2%) | 1324 (94.0%) | 386 (95.3%) | 391 (96.5%) | 1352 (96.0%) |  |
| Yes | 137 (3.8%) | 68 (4.8%) | 14 (3.5%) | 12 (3.0%) | 43 (3.1%) |  |
| Kidney disease |  |  |  |  |  | 0.671 |
| Missing | 56 (1.5%) | 19 (1.3%) | 6 (1.5%) | 3 (0.7%) | 28 (2.0%) |  |
| No | 3199 (88.2%) | 1241 (88.1%) | 360 (88.9%) | 360 (88.9%) | 1238 (87.9%) |  |
| Yes | 371 (10.2%) | 148 (10.5%) | 39 (9.6%) | 42 (10.4%) | 142 (10.1%) |  |
| Stomach or other digestive disease |  |  |  |  |  | 0.083 |
| Missing | 47 (1.3%) | 18 (1.3%) | 5 (1.2%) | 2 (0.5%) | 22 (1.6%) |  |
| No | 2380 (65.6%) | 961 (68.3%) | 268 (66.2%) | 261 (64.4%) | 890 (63.2%) |  |
| Yes | 1199 (33.1%) | 429 (30.5%) | 132 (32.6%) | 142 (35.1%) | 496 (35.2%) |  |
| Memory related disease |  |  |  |  |  | 0.564 |
| Missing | 32 (0.9%) | 11 (0.8%) | 4 (1.0%) | 1 (0.2%) | 16 (1.1%) |  |
| No | 3504 (96.6%) | 1367 (97.1%) | 388 (95.8%) | 393 (97.0%) | 1356 (96.3%) |  |
| Yes | 90 (2.5%) | 30 (2.1%) | 13 (3.2%) | 11 (2.7%) | 36 (2.6%) |  |
| Arthritis or rheumatism |  |  |  |  |  | 0.265 |
| Missing | 46 (1.3%) | 15 (1.1%) | 4 (1.0%) | 4 (1.0%) | 23 (1.6%) |  |
| No | 1917 (52.9%) | 737 (52.3%) | 199 (49.1%) | 230 (56.8%) | 751 (53.3%) |  |
| Yes | 1663 (45.9%) | 656 (46.6%) | 202 (49.9%) | 171 (42.2%) | 634 (45.0%) |  |
| Hearing impairment |  |  |  |  |  | 0.165 |
| Excellent | 51 (1.4%) | 15 (1.1%) | 6 (1.5%) | 9 (2.2%) | 21 (1.5%) |  |
| Fair | 1990 (54.9%) | 768 (54.5%) | 214 (52.8%) | 219 (54.1%) | 789 (56.0%) |  |
| Good | 571 (15.7%) | 219 (15.6%) | 76 (18.8%) | 61 (15.1%) | 215 (15.3%) |  |
| Missing | 526 (14.5%) | 229 (16.3%) | 55 (13.6%) | 65 (16.0%) | 177 (12.6%) |  |
| Poor | 488 (13.5%) | 177 (12.6%) | 54 (13.3%) | 51 (12.6%) | 206 (14.6%) |  |
| Pain |  |  |  |  |  | 0.855 |
| Missing | 46 (1.3%) | 20 (1.4%) | 5 (1.2%) | 3 (0.7%) | 18 (1.3%) |  |
| No | 2431 (67.0%) | 938 (66.6%) | 279 (68.9%) | 279 (68.9%) | 935 (66.4%) |  |
| Yes | 1149 (31.7%) | 450 (32.0%) | 121 (29.9%) | 123 (30.4%) | 455 (32.3%) |  |
| Weight change |  |  |  |  |  | 0.01 |
| Don't Know | 202 (5.6%) | 72 (5.1%) | 24 (5.9%) | 19 (4.7%) | 87 (6.2%) |  |
| Missing | 10 (0.3%) | 2 (0.1%) | 1 (0.2%) | 1 (0.2%) | 6 (0.4%) |  |
| No | 2906 (80.1%) | 1129 (80.2%) | 318 (78.5%) | 313 (77.3%) | 1146 (81.4%) |  |
| Yes, first gained and then lost weight | 33 (0.9%) | 9 (0.6%) | 3 (0.7%) | 7 (1.7%) | 14 (1.0%) |  |
| Yes, first lost and then gained weight | 24 (0.7%) | 11 (0.8%) | 3 (0.7%) | 4 (1.0%) | 6 (0.4%) |  |
| Yes, only gained weight | 159 (4.4%) | 78 (5.5%) | 17 (4.2%) | 28 (6.9%) | 36 (2.6%) |  |
| Yes, only lost weight | 292 (8.1%) | 107 (7.6%) | 39 (9.6%) | 33 (8.1%) | 113 (8.0%) |  |
| Health status during childhood |  |  |  |  |  | 0.012 |
| Excellent | 366 (10.1%) | 143 (10.2%) | 37 (9.1%) | 42 (10.4%) | 144 (10.2%) |  |
| Fair | 836 (23.1%) | 299 (21.2%) | 107 (26.4%) | 96 (23.7%) | 334 (23.7%) |  |
| Good | 650 (17.9%) | 254 (18.0%) | 63 (15.6%) | 89 (22.0%) | 244 (17.3%) |  |
| Missing | 1500 (41.4%) | 625 (44.4%) | 163 (40.2%) | 153 (37.8%) | 559 (39.7%) |  |
| Poor | 274 (7.6%) | 87 (6.2%) | 35 (8.6%) | 25 (6.2%) | 127 (9.0%) |  |
| Self expectations of health status |  |  |  |  |  | 0.357 |
| Almost certain | 468 (12.9%) | 186 (13.2%) | 50 (12.3%) | 61 (15.1%) | 171 (12.1%) |  |
| Almost impossible | 474 (13.1%) | 171 (12.1%) | 48 (11.9%) | 60 (14.8%) | 195 (13.8%) |  |
| Maybe | 1098 (30.3%) | 441 (31.3%) | 118 (29.1%) | 108 (26.7%) | 431 (30.6%) |  |
| Missing | 611 (16.9%) | 230 (16.3%) | 64 (15.8%) | 67 (16.5%) | 250 (17.8%) |  |
| Not very likely | 569 (15.7%) | 229 (16.3%) | 76 (18.8%) | 54 (13.3%) | 210 (14.9%) |  |
| Very likely | 406 (11.2%) | 151 (10.7%) | 49 (12.1%) | 55 (13.6%) | 151 (10.7%) |  |

This table provides the complete baseline covariate profile used for descriptive comparison across MCMI change-pattern groups.

**Table S3. Exposure profile and geographic coverage by two-wave MCMI change pattern group in the analytical cohort (2011–2018).**

| Characteristic | Overall (n=3626) | Stable high (n=1408) | High to low (n=405) | Low to high (n=405) | Stable low (n=1408) |
| --- | --- | --- | --- | --- | --- |
| Province |  |  |  |  |  |
| Missing | 4 (0.1%) | 4 (0.3%) | 0 (0.0%) | 0 (0.0%) | 0 (0.0%) |
| Anhui Province | 222 (6.1%) | 84 (6.0%) | 23 (5.7%) | 16 (4.0%) | 99 (7.0%) |
| Beijing Municipality | 1 (0.0%) | 1 (0.1%) | 0 (0.0%) | 0 (0.0%) | 0 (0.0%) |
| Fujian Province | 79 (2.2%) | 23 (1.6%) | 4 (1.0%) | 9 (2.2%) | 43 (3.1%) |
| Gansu Province | 90 (2.5%) | 37 (2.6%) | 8 (2.0%) | 9 (2.2%) | 36 (2.6%) |
| Guangdong Province | 67 (1.8%) | 14 (1.0%) | 9 (2.2%) | 6 (1.5%) | 38 (2.7%) |
| Guangxi Zhuang Autonomous Region | 96 (2.6%) | 22 (1.6%) | 9 (2.2%) | 7 (1.7%) | 58 (4.1%) |
| Guizhou Province | 40 (1.1%) | 4 (0.3%) | 8 (2.0%) | 4 (1.0%) | 24 (1.7%) |
| Hebei Province | 230 (6.3%) | 107 (7.6%) | 24 (5.9%) | 37 (9.1%) | 62 (4.4%) |
| Henan Province | 389 (10.7%) | 202 (14.3%) | 33 (8.1%) | 35 (8.6%) | 119 (8.5%) |
| Heilongjiang Province | 60 (1.7%) | 23 (1.6%) | 12 (3.0%) | 6 (1.5%) | 19 (1.3%) |
| Hubei Province | 76 (2.1%) | 24 (1.7%) | 7 (1.7%) | 5 (1.2%) | 40 (2.8%) |
| Hunan Province | 202 (5.6%) | 78 (5.5%) | 21 (5.2%) | 18 (4.4%) | 85 (6.0%) |
| Jilin Province | 53 (1.5%) | 17 (1.2%) | 6 (1.5%) | 10 (2.5%) | 20 (1.4%) |
| Jiangsu Province | 171 (4.7%) | 65 (4.6%) | 14 (3.5%) | 29 (7.2%) | 63 (4.5%) |
| Jiangxi Province | 179 (4.9%) | 50 (3.6%) | 23 (5.7%) | 21 (5.2%) | 85 (6.0%) |
| Liaoning Province | 100 (2.8%) | 40 (2.8%) | 7 (1.7%) | 19 (4.7%) | 34 (2.4%) |
| Inner Mongolia Autonomous Region | 146 (4.0%) | 65 (4.6%) | 21 (5.2%) | 22 (5.4%) | 38 (2.7%) |
| Qinghai Province | 55 (1.5%) | 21 (1.5%) | 9 (2.2%) | 2 (0.5%) | 23 (1.6%) |
| Shandong Province | 379 (10.5%) | 176 (12.5%) | 40 (9.9%) | 43 (10.6%) | 120 (8.5%) |
| Shanxi Province | 132 (3.6%) | 71 (5.0%) | 19 (4.7%) | 11 (2.7%) | 31 (2.2%) |
| Shaanxi Province | 143 (3.9%) | 44 (3.1%) | 15 (3.7%) | 16 (4.0%) | 68 (4.8%) |
| Shanghai Municipality | 5 (0.1%) | 2 (0.1%) | 1 (0.2%) | 1 (0.2%) | 1 (0.1%) |
| Sichuan Province | 321 (8.9%) | 102 (7.2%) | 50 (12.3%) | 28 (6.9%) | 141 (10.0%) |
| Tianjin Municipality | 33 (0.9%) | 20 (1.4%) | 3 (0.7%) | 6 (1.5%) | 4 (0.3%) |
| Xinjiang Uygur Autonomous Region | 12 (0.3%) | 9 (0.6%) | 0 (0.0%) | 2 (0.5%) | 1 (0.1%) |
| Yunnan Province | 188 (5.2%) | 51 (3.6%) | 19 (4.7%) | 25 (6.2%) | 93 (6.6%) |
| Zhejiang Province | 120 (3.3%) | 43 (3.1%) | 19 (4.7%) | 11 (2.7%) | 47 (3.3%) |
| Chongqing Municipality | 33 (0.9%) | 9 (0.6%) | 1 (0.2%) | 7 (1.7%) | 16 (1.1%) |
| MCMI_w1 | 2.91 [2.49, 3.42] | 3.53 [3.22, 3.96] | 3.11 [3.00, 3.28] | 2.70 [2.48, 2.81] | 2.43 [2.20, 2.65] |
| MCMI_w3 | 2.98 [2.53, 3.45] | 3.56 [3.26, 3.90] | 2.72 [2.50, 2.88] | 3.20 [3.09, 3.40] | 2.47 [2.23, 2.70] |
| dMCMI | 0.03 [-0.26, 0.31] | 0.02 [-0.30, 0.28] | -0.44 [-0.75, -0.23] | 0.56 [0.39, 0.86] | 0.03 [-0.17, 0.23] |
| MCMI_cum | 11.74 [10.08, 13.64] | 14.15 [13.21, 15.65] | 11.69 [11.25, 12.06] | 11.77 [11.30, 12.21] | 9.78 [8.91, 10.52] |

Continuous variables are summarized as median (IQR), and categorical variables as n (%).

This table summarizes MCMI-related exposure metrics across waves and province-level coverage to aid interpretation; baseline covariates with P values are provided in Table S2.

**Table S4. Exploratory subgroup analyses for two-wave MCMI change patterns and incident edentulism.**

| Stratum | Level | term | RR_CI | *p* |
| --- | --- | --- | --- | --- |
| Sex | Female | High to low | 0.644 (0.341, 1.217) | 0.175 |
| Sex | Female | Low to high | 0.843 (0.436, 1.629) | 0.611 |
| Sex | Female | Stable low | 0.925 (0.530, 1.615) | 0.785 |
| Sex | Male | High to low | 0.458 (0.179, 1.168) | 0.102 |
| Sex | Male | Low to high | 0.734 (0.256, 2.109) | 0.566 |
| Sex | Male | Stable low | 1.071 (0.495, 2.316) | 0.861 |
| Age | >=65 | High to low | 0.630 (0.302, 1.316) | 0.219 |
| Age | >=65 | Low to high | 0.251 (0.090, 0.700) | 0.008 |
| Age | >=65 | Stable low | 0.967 (0.509, 1.838) | 0.919 |
| Age | 45-55 | High to low | 0.596 (0.067, 5.256) | 0.641 |
| Age | 45-55 | Low to high | 3.201 (1.211, 8.461) | 0.019 |
| Age | 45-55 | Stable low | 0.965 (0.233, 3.995) | 0.961 |
| Age | 55-65 | High to low | 0.548 (0.224, 1.339) | 0.187 |
| Age | 55-65 | Low to high | 1.130 (0.476, 2.684) | 0.781 |
| Age | 55-65 | Stable low | 0.980 (0.501, 1.920) | 0.954 |

Model 3 RRs (95% CIs) are reported within prespecified subgroups. Stable high is the reference MCMI change-pattern group in each stratum.

**Table S5. Logistic regression sensitivity analysis for two-wave MCMI change patterns and incident edentulism.**

|  | |  | |  | | Model 1 | | | | Model 2 | | | | Model 3 | | |
| --- | --- | --- | --- | --- | --- | --- | --- | --- | --- | --- | --- | --- | --- | --- | --- | --- |
|  |  | Event (%) | | n | | OR (95% CI) | | *P* value | | OR (95% CI) | *P* value | | | OR (95% CI) | *P* value | |
| Stable high | 86 (6.1%) | | 1408 | | Reference | |  | | Reference | | |  | Reference | | |  |
| High to low | 17 (4.2%) | | 405 | | 0.61 (0.35, 1.06) | | 0.08 | | 0.58 (0.33, 1.03) | | | 0.062 | 0.56 (0.31, 1.00) | | | 0.049 |
| Low to high | 21 (5.2%) | | 405 | | 0.90 (0.54, 1.49) | | 0.674 | | 0.88 (0.52, 1.47) | | | 0.621 | 0.79 (0.43, 1.43) | | | 0.433 |
| Stable low | 107 (7.6%) | | 1408 | | 1.10 (0.75, 1.59) | | 0.633 | | 1.06 (0.72, 1.56) | | | 0.782 | 0.94 (0.57, 1.56) | | | 0.809 |

Odds ratios (ORs) and 95% confidence intervals (CIs) were estimated using logistic regression models.

Model 1 was adjusted for age, sex, geographic region (East, Central, West), smoking status, drinking status, and body mass index category;

Model 2 was further adjusted for education level, standard of living, and sleep duration;

Model 3 was additionally adjusted for baseline modified cardiometabolic index (MCMI). The stable high change pattern was used as the reference group.

**Table S6. Continuous MCMI summaries and incident edentulism.**

| Exposure | Scale | Model | N | Events | RR (95% CI) | P value |
| --- | --- | --- | --- | --- | --- | --- |
| MCMI_w1 | per 1 SD increase | Model 1 | 3626 | 231 | 0.94 (0.80, 1.11) | 0.478 |
| MCMI_w1 | per 1 SD increase | Model 2 | 3626 | 231 | 0.93 (0.79, 1.10) | 0.422 |
| MCMI_w1 | per 1 SD increase | Model 3 | 3626 | 231 | 0.93 (0.79, 1.10) | 0.422 |
| MCMI_w3 | per 1 SD increase | Model 1 | 3626 | 231 | 1.01 (0.86, 1.17) | 0.937 |
| MCMI_w3 | per 1 SD increase | Model 2 | 3626 | 231 | 1.00 (0.86, 1.17) | 0.969 |
| MCMI_w3 | per 1 SD increase | Model 3 | 3626 | 231 | 1.04 (0.89, 1.22) | 0.635 |
| dMCMI | per 1 SD increase | Model 1 | 3626 | 231 | 1.06 (0.93, 1.20) | 0.365 |
| dMCMI | per 1 SD increase | Model 2 | 3626 | 231 | 1.07 (0.94, 1.21) | 0.325 |
| dMCMI | per 1 SD increase | Model 3 | 3626 | 231 | 1.04 (0.88, 1.24) | 0.635 |
| MCMI_cum | per 1 SD increase | Model 1 | 3626 | 231 | 0.97 (0.82, 1.14) | 0.683 |
| MCMI_cum | per 1 SD increase | Model 2 | 3626 | 231 | 0.96 (0.81, 1.13) | 0.624 |
| MCMI_cum | per 1 SD increase | Model 3 | 3626 | 231 | 1.08 (0.79, 1.47) | 0.635 |

Values are risk ratios (RRs) with 95% confidence intervals estimated per 1-SD increase in each continuous MCMI summary. Model 1 adjusted for age, sex, region, smoking status, drinking status, and body mass index category. Model 2 additionally adjusted for education level, standard of living, and sleep duration. Model 3 further adjusted for baseline MCMI where applicable. MCMI, modified cardiometabolic index.

**Table S7. Multiple-imputation sensitivity analysis for MCMI change patterns and incident edentulism.**

| Model | Reference | MCMI change pattern | RR (95% CI) | P value |
| --- | --- | --- | --- | --- |
| Model 3 with multiple imputation for covariates | Stable high | High to low | 0.57 (0.34, 0.96) | 0.036 |
| Model 3 with multiple imputation for covariates | Stable high | Low to high | 0.79 (0.47, 1.33) | 0.378 |
| Model 3 with multiple imputation for covariates | Stable high | Stable low | 0.91 (0.60, 1.36) | 0.631 |

Reference group is stable high MCMI. Multiple imputation by chained equations was used for covariates with missing values; exposure group and outcome status were not imputed. Estimates are based on the Model 3 covariate structure and are presented as RR (95% CI).

**Table S8. Baseline characteristics of included participants and those excluded after the baseline non-edentulous risk set.**

| **Variable** | **Excluded after baseline non-edentulous risk set (n=10401)** | **Included final analytic cohort (n=3626)** | **Absolute SMD** |
| --- | --- | --- | --- |
| Age |  |  | 0.188 |
| >=65 | 3092 (29.7%) | 1130 (31.2%) |  |
| 45-55 | 3548 (34.1%) | 925 (25.5%) |  |
| 55-65 | 3761 (36.2%) | 1571 (43.3%) |  |
| Sex |  |  | 0.127 |
| Female | 5195 (49.9%) | 2040 (56.3%) |  |
| Male | 5206 (50.1%) | 1586 (43.7%) |  |
| Regional |  |  | 0.095 |
| Central | 3658 (35.2%) | 1442 (39.8%) |  |
| East | 3683 (35.4%) | 1185 (32.7%) |  |
| Missing | 1 (0.0%) | 4 (0.1%) |  |
| West | 3059 (29.4%) | 995 (27.4%) |  |
| Education level |  |  | 0.091 |
| Elementary school | 2301 (22.1%) | 809 (22.3%) |  |
| High school or above | 1409 (13.5%) | 384 (10.6%) |  |
| Less than elementary school | 4314 (41.5%) | 1649 (45.5%) |  |
| Middle school | 2377 (22.9%) | 784 (21.6%) |  |
| Standard of living |  |  | 0.260 |
| Average | 2769 (26.6%) | 1272 (35.1%) |  |
| Missing | 5645 (54.3%) | 1497 (41.3%) |  |
| Poor | 667 (6.4%) | 279 (7.7%) |  |
| Relatively high | 311 (3.0%) | 102 (2.8%) |  |
| Relatively poor | 968 (9.3%) | 455 (12.5%) |  |
| Very high | 41 (0.4%) | 21 (0.6%) |  |
| Sleeping time |  |  | 0.246 |
| >8 h | 918 (8.8%) | 334 (9.2%) |  |
| ≤6 h | 4843 (46.6%) | 1846 (50.9%) |  |
| 6–8 h | 3845 (37.0%) | 1363 (37.6%) |  |
| Missing | 795 (7.6%) | 83 (2.3%) |  |
| Smoking Status |  |  | 0.100 |
| Missing | 52 (0.5%) | 0 (0.0%) |  |
| No | 5604 (53.9%) | 2133 (58.8%) |  |
| Yes | 4745 (45.6%) | 1493 (41.2%) |  |
| Drinking Status |  |  | 0.060 |
| Missing | 85 (0.8%) | 13 (0.4%) |  |
| No | 6621 (63.7%) | 2409 (66.4%) |  |
| Yes | 3695 (35.5%) | 1204 (33.2%) |  |
| BMI category clean |  |  | 0.768 |
| Missing | 2766 (26.6%) | 13 (0.4%) |  |
| Normal weight | 3694 (35.5%) | 1675 (46.2%) |  |
| Obese | 1006 (9.7%) | 513 (14.1%) |  |
| Overweight | 2487 (23.9%) | 1265 (34.9%) |  |
| Underweight | 448 (4.3%) | 160 (4.4%) |  |
| Hypertension |  |  | 0.207 |
| Missing | 607 (5.8%) | 67 (1.8%) |  |
| No | 6583 (63.3%) | 2244 (61.9%) |  |
| Yes | 3211 (30.9%) | 1315 (36.3%) |  |
| Dyslipidaemia |  |  | 0.178 |
| Missing | 851 (8.2%) | 143 (3.9%) |  |
| No | 7820 (75.2%) | 2698 (74.4%) |  |
| Yes | 1730 (16.6%) | 785 (21.6%) |  |
| Diabetes |  |  | 0.188 |
| Missing | 677 (6.5%) | 94 (2.6%) |  |
| No | 8766 (84.3%) | 3150 (86.9%) |  |
| Yes | 958 (9.2%) | 382 (10.5%) |  |
| Heart disease |  |  | 0.186 |
| Missing | 619 (6.0%) | 82 (2.3%) |  |
| No | 8036 (77.3%) | 2875 (79.3%) |  |
| Yes | 1746 (16.8%) | 669 (18.5%) |  |
| Stroke |  |  | 0.256 |
| Missing | 578 (5.6%) | 36 (1.0%) |  |
| No | 9443 (90.8%) | 3453 (95.2%) |  |
| Yes | 380 (3.7%) | 137 (3.8%) |  |
| MCMI w1 | 2.86 [2.42, 3.37] | 2.91 [2.49, 3.42] | 0.071 |
| MCMI w3 | 2.93 [2.49, 3.40] | 2.98 [2.53, 3.45] | 0.034 |

Values are n (%) for categorical variables and median [interquartile range] for continuous variables. Absolute standardized mean differences (SMDs) compare participants included in the final analytic cohort with those excluded after the baseline non-edentulous risk set. Edentulism status at 2015 and 2018 was not displayed in this table because these variables were part of subsequent eligibility and outcome ascertainment rather than baseline characteristics.

**Figure S1. Continuous MCMI summaries and incident edentulism.**

Spline-based associations of MCMI in 2015, change in MCMI from 2011 to 2015, and cumulative MCMI with incident edentulism. Models used the Model 2 covariate structure. Risk ratios are referenced to the median value of each exposure, indicated by the vertical dotted line. Shaded areas indicate 95% confidence intervals. P values are for the overall spline association.
